# Information and communication technology in disease surveillance, India: a case study

**DOI:** 10.1186/1471-2458-10-S1-S11

**Published:** 2010-12-03

**Authors:** Lalit Kant, Sampath K Krishnan

**Affiliations:** 1 Division of Epidemiology & Communicable Diseases, Indian Council of Medical Research, Ansari Nagar, New Delhi 110029, India; 2 WHO Country Office for India, Nirman Bhawan, New Delhi 110011, India

## Abstract

India has made appreciable progress and continues to demonstrate a strong commitment for establishing and operating a disease surveillance programme responsive to the requirements of the International Health Regulations (IHR[2005]). Within five years of its launch, India has effectively used modern information and communication technology for collection, storage, transmission and management of data related to disease surveillance and effective response. Terrestrial and/or satellite based linkages are being established within all states, districts, state-run medical colleges, infectious disease hospitals, and public health laboratories. This network enables speedy data transfer, video conferencing, training and e-learning for outbreaks and programme monitoring. A 24x7 call centre is in operation to receive disease alerts. To complement these efforts, a media scanning and verification cell functions to receive reports of early warning signals. During the 2009 H1N1 outbreak, the usefulness of the information and communication technology (ICT) network was well appreciated. India is using ICT as part of its Integrated Disease Surveillance Project (IDSP) to help overcome the challenges in further expansion in hard-to-reach populations, to increase the involvement of the private sector, and to increase the use of other modes of communication like e-mail and voicemail.

## Background

With the adoption of the International Health Regulations [IHR(2005)] by the World Health Organization (WHO), it has become obligatory for the Member States to put in place requisite manpower, money and material to comply with the provisions of the regulation. Each signatory country is expected to achieve identified capabilities, including developing and maintaining core capacities to detect and respond to potential public health emergencies of international concern (PHEIC) within its national borders. Use of modern tools of communication and international technology has helped to scale-up the rapid sharing of data.

For rapid detection and response to any outbreak, natural or deliberate, a sensitive surveillance system is essential. A good communication system is the ‘brain’ of the surveillance system. It is the speed of communication which is most critical to contain or stamp out an outbreak, save lives, and prevent misery. It is challenging, however, to set up an effective communication system, and even more so in a country as large, populous, and technologically and linguistically varied as India.

India, with an estimated population of 1.15 billion in 2010, supports almost one fifth of world’s population on only about 2.5 percent of its surface area [1]. India adds on an average about 22 million newborns to its population every year [1]. Population density is also high, with a national average of 312 people per square kilo metre [2]. India has a very diverse population, speaking 325 recognized languages, making communication extremely challenging [3]. This ever-growing population is spread out over 28 states and 7 Union Territories ruled by parties with varying ideologies and developmental agenda. The Constitution of India provides autonomy to each state (federal structure), and lists out their responsibilities. Health has been identified as a state subject. There is thus a differential growth pattern of setting up and utilization of disease surveillance infrastructure. Under the health system there are a large number of disease control programmes, each with its own system of data gathering. Historically, these have been vertical programmes. With the launch of the Integrated Disease Surveillance Project in 2004, an initiative has been undertaken to bring many of them under one roof [4].

## Integrated Disease Surveillance Project

The Union Ministry of Health & Family Welfare adopted an Integrated Disease Surveillance Project (IDSP) to connect all district hospitals and state-run medical colleges to facilitate tele-education, training of health professionals and monitoring of disease trends. The disparity between states’ urban and rural regions is bridged using modern information and communication technology (ICT). The goal of this World Bank-assisted project (USD 102 million), is to establish a decentralized, state-based disease surveillance system. The project aims to detect early warning signals of impending disease outbreaks and initiate effective and rapid health actions with the help of ICT at the district, state and national levels. The project is implemented through the Central Surveillance Unit within the National Centre for Disease Control (NCDC) at New Delhi [4].

The IDSP is being strengthened to make it compliant with IHR(2005) requirements by supporting a nationwide effort for the immediate reporting of outbreaks. The establishment and operation of decentralized surveillance system meeting performance standards, with technical support from the WHO and the U.S. Centers for Disease Control and Prevention (CDC) in the areas of disease surveillance and outbreak investigations, health informatics, and laboratory-based surveillance, has been vital in implementation of IHR(2005). Several specific steps have been taken to:

1) enhance the quality and frequency of use of communication network;

2) further training for district level epidemiologists and data managers on use of portal;

3) streamline the urban surveillance component;

4) enhance networking of infectious disease hospitals;

5) improve quality and timeliness of reporting.

### Components of the IDSP

Some of the key components of the IDSP in India include:

• Integration and decentralization of surveillance activities through establishment of surveillance units at national, state and district levels;

• Human Resource Development through training of State Surveillance Officers, District Surveillance Officers, multi-disciplinary Rapid Response Teams, and other medical and paramedical staff on all aspects of disease surveillance;

• Use of ICT for the collection, collation, compilation, analysis and dissemination of data through a web portal;

• Strengthening of public health laboratories.

In addition to monitoring diseases like vector borne infections, diarrhoeal diseases, respiratory diseases, and vaccine preventable diseases, the IDSP also has a category called ‘unusual clinical syndromes,’ which would alert public health professionals to an intentional release of any biological-agent. The local governments in each state have the option to add any other health condition to the list as deemed appropriate. The ICT network is then used for the collection, analysis, and rapid communication of data.

### Structure of the ICT network

Eight hundred sites have been identified to be part of an ICT satellite network. Sites include District Headquarters (604), State Headquarters (35), academic institutions like Government Medical Colleges (133), premier medical institutions (15) and Infectious Disease Hospitals (7). At the national level these are connected to the National Informatics Centre, the Central Surveillance Unit (at the NCDC) and the Ministry of Health & Family Welfare. [5]

### Infrastructure

The India Space Research Organization (ISRO), through its Education Satellite (EDUSAT), has connected 360 of the 400 ICT network sites. The network operation centre (the principal teaching end) is housed at the NCDC, and the Satellite Interactive Terminals (SITs) are located at State Headquarters, District Surveillance Units and Government Medical Colleges. ISRO connects all sites in northeastern states, hilly and island states, Tamil Nadu, Gujarat, and Maharashtra. The National Informatics Centre (NIC) has provided terrestrial (broadband) connectivity to 776 of the 800 sites. It has also provided high-end video conference equipments at all state and Union Territory headquarters. Upon completion, the network will enable 800 sites on a broadband network of which 400 sites will have dual connectivity with satellite and broadband [5,6].

### IDSP portal

The NIC has created a single-stop web portal (http://www.idsp.nic.in) with options for data entry and analysis from the district level upwards related to disease surveillance. It is quicker, easier, and more powerful to update than print-based media. The portal can also convert data into charts and graphs. The data entry operators and data managers have been trained in data entry, analysis and transmission. The portal also has information on media and outbreak alerts.

### Media Scanning and Verifcation Cell

India has a very vibrant and active media. There are close to 70,000 newspapers (as of 31 March 2008) [7] and over 500 television channels [8]. Media is therefore a very important source of information and its power is now being harnessed to detect outbreaks. A Media Scanning and Verification Cell was established at the NCDC in July 2008. This cell collates reported unusual health events on infectious diseases within the country and informs the concerned state, district, and national level health officials. These are then investigated and verified. Verified events are conveyed to appropriate authority for further action. About a thousand health alerts have been detected since July 2008. This process increases the sensitivity of the official surveillance system, and may provide early warning of occurrences of new clusters of diseases in advance of official notification, as the media is often the first to know of potential cases [4].

### Call centre

The telecommunication industry is one of the fastest growing industries in India. The total telephone subscription base is approaching 600 million [8]. The overall tele-density is over 50 percent, and urban tele-density has reached 100 percent. Subscriptions are growing by nearly 20 million new subscribers per month. Tele-density is expected to go up to 84 percent by 2012 [1]. Ready access to telephone systems by the general population is rapidly increasing. Citizens themselves could be a good source of disease outbreak information. With this in mind, a 24x7 call centre was established in February 2008 with a toll free number (1075) to receive disease alerts. The call centre has multiple language calling and answering capabilities, and any person speaking one of the major regional languages is welcome to call in with information about outbreaks/unusual events. The information is relayed to the appropriate District or State Level Surveillance Units, as well as to Central Outbreak Monitoring Cell at the IDSP, which monitors the action taken by the concerned district. Since its inception, more than 200,000 calls have been received, and more than 150 were confirmed health alerts [5,6].

### Mechanism of data collection and transmission

Data is collected and reported on a weekly basis under the IDSP. When health workers at sub-centres suspect a case, they fill out Form-S (suspect case). The doctors at Primary Health Centres, Community Health Centres and Hospitals fill form P (probable case/presumptive case). The cases that are confirmed by the laboratory fill up Form L (lab-confirmed case). Data managers receive these completed forms from various centres, and data compilation and analysis is done at the district, state and national levels. Cases representing and unusual or rising trends are investigated by a Rapid Response Team (RRT) to diagnose and control the outbreak. Major hospitals and Infectious Disease Hospitals are also required to report surveillance data. Eighty-five percent of all districts are now reporting weekly data to IDSP [5,6].

### Video conferencing

To ensure preparedness and enhance the quality of disease surveillance, regular video conferences are organized by central surveillance unit of IDSP to discuss outbreak investigations and verification and control measures with various State Surveillance Officers, District Surveillance Officers and the Rapid Response Teams. Additionally, project review and monitoring for different aspects of the IDSP is undertaken periodically. Video conferencing facilities are also used for training purposes [5,6].

### Distance learning

For efficient functioning of the surveillance system training of various IDSP functionaries is an on-going process. Educational satellite (EDUSAT) classrooms are available at State Headquarters, District Headquarters, Medical Colleges, Premier Institutions and Infectious Disease Hospitals [5,9]. The communication network is also used to manage live virtual classrooms for training, e-learning, and interactive electronic discussions, among other things. Training for data managers and data entry operators and refresher courses and continuing education sessions are held regularly. Further training of epidemiologists and data managers can help build analytic capacity at district levels. Appropriate e-modules have been developed. Scanners have been provided to convert the x-ray pictures and paper reports into soft copies so that they could be electronically transmitted and also used for teaching purposes.

### Use of communication network during the H1N1 pandemic

The call centre received 35,000 calls related to H1N1. The video conferencing facilities were used for training of medical officers in clinical management, infection control practices and laboratory support. The network also facilitated prompt receipt of test results.

## Conclusions

Appreciable progress has been made in putting together the infrastructure and development of skills to make the ICT network functional in disease surveillance programme. As previously mentioned, 776 out of 800 sites have been networked, 738 sites have videoconferencing facilities, about 850,000 sub-centres send weekly report on syndromes, about 150,000 Primary Health Centres, Community Health Centres, and hospitals send list of probable cases. Facilities for distance education have also been established. There are other areas which still need to be addressed, including encouraging private sector reporting, strengthening skills for data use and analysis at all levels, enhancing the convergence of information and communication technology with other national health programmes, improving media scanning and verification, and making data transmission all-inclusive by incorporating, mobile, text, voice, email and fax.

## Abbreviations

CDC: Centers for Disease Control and Prevention; EDUSAT: Education Satellite; ICT: Information and Communication Technology; IDSP: Integrated Disease Surveillance Project; ISRO: Indian Space Research Organization; NCDC: National Centre for Disease Control; NIC: National Informatics Centre; PHEIC: Public Health Emergencies of International Concern; SIT: Satellite Interactive Terminals; WHO: World Health Organization; IHR: International Health Regulations.

## Competing interests

The authors declare that they have no competing interests.

## Authors’ contributions

LK: developed the concept, drafted and finalized the manuscript. SKK: provided information, revised the manuscript.

Both authors read and approved the manuscript.
